# Is Physical Rehabilitation Need Associated With the Rehabilitation Workforce Supply? An Ecological Study Across 35 High-Income Countries

**DOI:** 10.34172/ijhpm.2020.150

**Published:** 2020-08-17

**Authors:** Tiago S. Jesus, Michel D. Landry, Helen Hoenig, Gilles Dussault, Gerald C. Koh, Inês Fronteira

**Affiliations:** ^1^Global Health and Tropical Medicine & WHO Collaborating Center on Health Workforce Policy and Planning, Institute of Hygiene and Tropical Medicine - NOVA University of Lisbon, Lisbon, Portugal.; ^2^School of Medicine, Duke University, Durham, NC, USA.; ^3^Duke Global Health Institute, Duke University, Durham, NC, USA.; ^4^Physical Medicine and Rehabilitation Service, Durham Veterans Administration Medical Center, Durham, NC, USA.; ^5^Division of Geriatrics, Department of Medicine, Duke University Medical Center, Durham, NC, USA.; ^6^Saw Swee Hock School of Public Health, National University of Singapore, Singapore, Singapore.

**Keywords:** Health Workforce, Rehabilitation, Physical Therapy, Occupational Therapy, Health Services Needs, High-Income Countries

## Abstract

**Background:** To determine whether population-adjusted rates of physical rehabilitation need (ie, disability-related epidemiological data) are associated with the workforce supply (ie, combined rates of practicing physical therapists (PTs) and occupational therapists (OTs) per 10 000 population) across high-income countries (HICs), adjusted for socio-demographic and economic covariates.

**Methods: **This is a cross-national ecological study. Hierarchical, multiple linear regressions analyzed current international data across 35 HICs using: current PTs and OTs supply data obtained from the international professional federations (outcome variable); needs data obtained from the Global Burden of Disease 2017 (GBD 2017); and finally relevant socio-demographic variables and supply-side covariates extracted from the World Bank, GBD 2017, the supply data sources, and the Global Health Expenditure Database.

**Results: **The PTs and OTs per capita varied greatly across the 35 HICs, differing by as much as 40-fold. Denmark had the greatest supply per capita. Physical rehabilitation need was not a significant, independent predictor of workforce supply regardless of the multiple regression model used (*P* >.10). In the final model, after Bonferroni correction, 3 covariates were significant, independent predictors of the supply variable: gross national income (GNI) per capita and the current health expenditure in % of gross domestic product (GDP) were positive factors for workforce supply, while population size was a negative factor (all *P* <.01).

**Conclusion:** PT and OT workforce supply is highly variable across HICs. This variability is not accounted for by an indicator of population need but rather by financial indicators and population size.

## Background

**Key Messages**

Implications for policy makers

- Physical therapist (PT) and occupational therapist (OT) workforce supply is highly variable across high-income countries (HICs), and that was not accounted for by a composite indicator of population need, in contrast with financial and population size factors. These findings suggest substantial supply-need disparities across HICs. Deliberate, data-based physical rehabilitation workforce policies and planning should be developed as a result.
- Population size and, marginally, the percentage of rural population were two factors associated to the PT and OT workforce supply. People living in rural areas in countries with a larger population, may be facing a double disparity, or double ecological risk, of facing needs-based shortages of PTs and OTs, if explicit policies are not in place.
- Ecological findings need to be complemented with country-specific data and analyses, as not all countries (eg, Singapore) fitted the ecological trend identified. Countries with trends identified as at the odds with the ecological trend may be at a more pressing need for implementing deliberate workforce policies.

Implications for the public

Large physical therapist (PT) and occupational therapist (OT) workforce supply disparities were found across the 35 high-income countries (HICs), for relatively equivalent levels of population need. While population need was not significantly, independently associated with the PT and OT supply, population size and two financial factors were: gross national income (GNI) per capita, and the current health expenditure in % of gross domestic product (GDP). Altogether, while demand-side factors were associated to PT and OT supply, needs-based were not. Items on government spending in healthcare were not significant factors in this study. Yet, the five Nordic countries were within the top six in the PT and OT supply. The use of composite indicator of physical rehabilitation need, derived from the Global Burden of Disease 2017 (GBD 2017) and available since 2019, can be used in rehabilitation workforce supply and requirements studies, in addition or in complement to demand-based factors. Uncovering supply-need disparities across HICs may help drive further, deliberate policies to close them.

The determination of the health and rehabilitation workforce supply (here the number of health workers a country has in practicing roles) should account for the population need (here defined as relevant epidemiological data) for these services.^1-5^ This study aims to test whether current physical rehabilitation need is independently associated with current rehabilitation workforce supply in high-income countries (HICs).

 Deliberate needs-based workforce policies, including planning and research, are needed to ensure the equitable accessibility of health workers to the population.^1,2,5-10^ Global population ageing and higher rates of chronic conditions, disabilities and physical rehabilitation needs are growing in tandem.^11-13^ Since 1990, there has been a significant growth of physical rehabilitation needs per capita worldwide, including a 16% growth in HICs (*P *<.01).^14^ However, the rehabilitation workforce remains an understudied and often neglected health workforce.^3,15^ Furthermore, imbalances in the rehabilitation workforce supply are present among countries, even in HICs, with large variations observed.^3,15-17^ For example, in 2014, Singapore had a number of physical therapists (PTs) per capita 4.3 times lower than Portugal’s, even though the gross domestic product (GDP) was over than 3 times higher.^16^

 The ‘right’ size of the health and rehabilitation workforce must not be determined solely by ratios of providers per population, but must account for other variables, including population needs.^3,5,18-20^ It is poorly known whether the physical rehabilitation ‘needs’ of the population are a key determinant of its supply. Gupta et al^15^ used descriptive statistics and simple regression models to compare supply of and need for the rehabilitation workforce. However, this seminal research is based on data from low-income countries to HICs up to 2008. Their study did not adjust the findings for relevant socio-economic and health expenditures data, and it used years of life lost to assess rehabilitation needs – a metric of the Global Burden of Disease (GBD) study that emphasizes premature mortality outcomes. More recent, disability-focused and reliable data for determining rehabilitation needs and supply are currently available,^3,14^ and can be used alongside other relevant health and socio-economic indicators to understand the determinants of the rehabilitation workforce supply.

 This study focusses on HICs, where rehabilitation workforce data are more available and more robust and reliable, hence with greater comparability across nations.^3^ In HICs, the developments of rehabilitation services and workforce are not in its infancy, thereby an alignment among the rehabilitation workforce supply and need is much more likely and expected – and can be tested against that hypothesis. This contrasts with the many low- and middle-income countries (LMICs), where rehabilitation needs are known to be largely unmet^3,11,15^; for instance, the World Health Organization (WHO) estimated that qualified rehabilitation professionals are about one-tenth of those required in many LMICs.^11^ Nonetheless, whether unmet rehabilitation needs or needs-based inequalities in workforce supply exist among HICs is still unclear.

 Hence, with a focus on HICs, this paper aims to test the hypothesis that physical rehabilitation need, based on disability-focused data. ie, years lived with disability (YLDs) from the GBD 2017 is independently associated with physical rehabilitation workforce supply (ie, the combined number of practicing PTs and occupational therapists (OTs) 10 000 population), ie, after adjusting for socio-economic and demographic variables.

## Methods

 This is a cross-national, ecological study, using global health, workforce, and socio-demographic data available in the public domain.

###  Measures, Sources and Data Extraction

 The *dependent* (ie, outcome) variable is the physical rehabilitation workforce supply, which here is the population-adjusted combined number of practicing PTs and OTs, provided by the international professional federations.^21,22^ To compute this variable, we summed up the number of practicing PTs and OTs per 10 000 population for each HIC, according to the World Bank’s Classification of 2019. Data on the numbers of practicing PTs and OTs were retrieved from databases of the World Confederation for Physical Therapy (now known as World Physiotherapy), and the World Federation of Occupational Therapists.^21,22^ Both federations have active human resources projects mapping these professionals across countries, using best-available data, collected by the national professional associations, under a standard international procedure.^21,22^ Hence, we had reliable, internationally comparable supply data for these two rehabilitation professions whose scope of practice partly overlap.^16^ We consulted the WHO’s Global Health Workforce Statistics database (https://www.who.int/hrh/statistics/hwfstats7en/) and observed that the existing data for rehabilitation professions were less up to date, from variable sources and timings, and less comparable across countries. The supply data, extracted in July 2019, is current to November 2, 2017 for practicing OTs,^22^ and to June 30, 2018 for practicing PTs.^21^ We used the country population estimates from the GBD 2017 as the denominator for the ratio.^23^

 The main *independent* variable is the total amount, as of 2017, of physical rehabilitation ‘needs,’ adjusted for population size. To compute this variable, YLD rates per 10 000 population were extracted from the GBD 2017 for each rehabilitation-sensitive health condition, and then summed up to provide a composite indicator of physical rehabilitation need per country.^14,24^ YLDs is the aggregate measure of the GBD study that focusses exclusively on non-fatal health losses; it is computed using the prevalence of health conditions, the time people typically live with sequalae from those conditions, and a disability weight for the severity level.^24^ All these factors are relevant for determining physical rehabilitation needs, especially when combined into one measure. For instance, spinal cord injuries are not as prevalent as ankle sprains, but more often leave long-term sequalae, can be severe, require intensive physical rehabilitation, and for longer periods of time. In this example, relative to prevalence, YLDs are less prone to overestimate the need for physical rehabilitation from ankle sprains as well as less prone to underestimate physical rehabilitation needs arising from spinal cord injuries. Overall, YLDs is a more balanced measure of rehabilitation need across health conditions when compared to prevalence.

 To determine which YLDs in the rate metric were relevant to physical rehabilitation, we used the set of conditions which were determined as responsive to physical rehabilitation interventions.^14^Box 1 details that full set of conditions used to build the composite indicator of physical rehabilitation need from the GBD 2017. We then summed the 2017 YLD Rates for all conditions listed into Box 1, for each HIC.

**Box 1. Health Conditions (From the GBD Study 2017) Whose Summed YLD Rates Provide a Composite Indicator of Physical Rehabilitation Need, According to Jesus et al^14^**

** Causes**

Communicable, Maternal, Neonatal or Nutritional:

- HIV/AIDs
- Leprosy; Zika
- Meningitis, Encephalitis; Tetanus
- Neonatal disorders

Non-communicable

- Neoplasms
- Cardiovascular diseases (includes stroke)
- Chronic respiratory diseases
- Neurological disorders, except epilepsy and migraine (tension-type headaches included)
- Autism spectrum disorder
- Musculoskeletal conditions (includes low back pain and neck pain)
- Congenital birth defects, except urogenital and digestive

** Injuries (nature of the)**

- Amputations
- Burns
- Fractures, except skull
- Head injuries
- Spinal injuries
- Minor injuries: muscle and tendon injuries; including sprains and strains lesser dislocations; Open wound(s)
- Dislocation of hip; Dislocation of knee; and Dislocation of shoulder
- Asphyxiation
- Crush injury; Nerve injury; Severe chest injury
- Multiple fractures; dislocations; crashes; wounds; pains; and strains

** Impairments (from the non-selected “causes” combined)**

- Heart failure
- Guillain-Barré syndrome

Abbreviations: GBD, Global Burden of Disease; YLD, year lived with disability.

Table 1in turndetails all *covariates* for which we collected data on countries’ socio-economic and demographic variables that could affect, theoretically, the ‘demand’ for rehabilitation services above and beyond need.

**Table 1:** Covariates, by Type, for Which Data Was Collected and Used Into the Analysis

**Socio-Economic**
Country’s income – Macro indicator: *Nominal GNI per capita – 2017*, determined by the World Bank.^a^
Development status – Composite indicator: *Socio-demographic index (value), *from the GBD 2017 study.^b^
Country’s spending on health, as % of the GDP: *Current health expenditure as % of the GDP (2016)*, from the Global Health Expenditure Database.
Government spending on health, using three metrics from the Global Health Expenditure Database: *Domestic general government health expenditure as % general government expenditure (2016).* *Domestic general government health expenditure as % current health expenditure (2016).* *Domestic general government health expenditure as % GDP (2016).*
Government spending in the whole country’s economy: *General government expenditure as % of GDP*, extracted from the Global Health Expenditure Database.
*Rationale*: Income level, health spending, and the percentage of the health expenditures from national governments can affect the demand for health and rehabilitation workers.^8,19,25,26^ National governments accountable for a higher share of health costs more directly influence demand for rehabilitation workers, for greater or lower. For example, through adding to or restraining recruitment in public-run facilities or through a more or less expanded public reimbursement, or subsidized coverage, for rehabilitation services even in private facilities.^16^
**Demographic**
Population amount: *Population size (2017)*, extracted from GBD 2017 – population estimates.
Rural population *Percentage of Rural Population (2017*), using World Bank’s data.
*Rationale*:The countries’ population and the percentage of the population living in rural areas can interfere with workforce numbers, per-capita.^3,6,27^
**Supply-Side Covariates (Categorial)**
Authoritative source of PT supply data, yes or no: Whether the PT supply data were based on an authoritative source or no (estimate). Of note, there is no such data available for OTs.
PT assistants part of the workforce, yes or no: Whether PT support personnel officially (ie, legally) exist in the countries.
OT assistants part of the workforce, yes or no: Whether OT support personnel officially (ie, legally) exist in the countries.
*Rationale*:Supply-side covariates, categorial in nature, on whether authoritative or estimated sources were used for supply determination in each country (as a means to testing whether results are affected by the data source of the outcome variable), and whether assistant therapists, apart from PTs and OTs, existed officially in the country, as the existence of assistant professionals can also affect demand.^16,28^

Abbreviations: GNI, gross national income; GBD, Global Burden of Disease; GDP, gross domestic product; PT, physical therapist; OT, occupational therapist. ^a^The GNI measures all income of a country’s residents and businesses, regardless of where it is produced. This means that GNI is given by the GDP plus wages, salaries, property income, and subsidies of the country’s residents earned abroad. ^b^ This metric is computed using data on fertility, education and lag distributed outcome per capita, and strongly correlated with health outcomes. Note: The supply-side covariates are extracted from the same sources of the supply data – the outcome variable.^21,22^

 This metric is computed using data on fertility, education and lag distributed outcome per capita, and strongly correlated with health outcomes. Note: The supply-side covariates are extracted from the same sources of the supply data – the outcome variable.^21,22^

###  Countries

**Figure 1:**
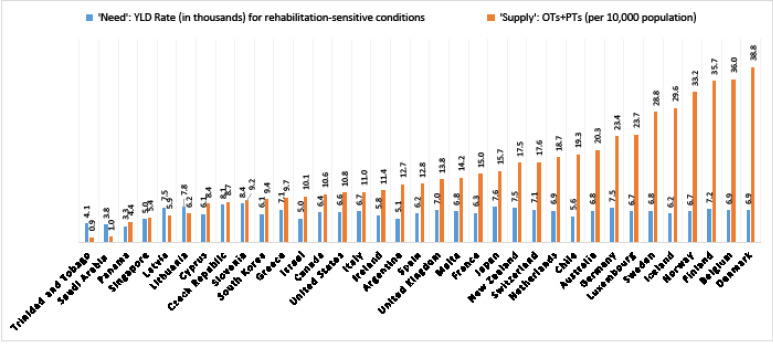
Physical Rehabilitation Need and Supply Indicators for Each of the 35 High-Income Countries Included. Abbreviations: PTs, physical therapists; OTs, occupational therapists; YLD, year lived with disability.

 We collected the last available data for a total of 37 HICs with supply data for PTs and OTs. Portugal and Estonia only had PT supply data up to 2015, and Switzerland up to 2017; all others had data up to 2018. We ran pilot analyses with and without Portugal and Estonia, and then with or without these and Switzerland as well. As the statistical significance of the main results (ie, independent, predictive value of the need indicator on workforce supply) was no different, we excluded the data from Portugal and Estonia but kept that of Switzerland – as the date was similar to other included countries (ie, 2017 and 2018, respectively). Hence, we included current supply data for 35 countries, including Switzerland, into our final analysis. Figure 1 (in the results section) depicts the 35 HICs, while theSupplementary file 1 provides the raw data for each.

###  Statistical Analysis

 We conducted a simple linear regression for the unadjusted effect of the main independent variable (need indicator) on the outcome variable (PTs and OTs rates). Then, we built a multiple linear regression with all covariates included. Categorical covariates, whose qualitative value were *yes* or *no*, were transformed into ‘dummy’ variables of values *1 *and *0, *respectively.

 Subsequently, to build a model containing only the covariates that were found relevant for the data, we started by eliminating all the covariates that had no significant relationship with the outcome variable (*P* values >.05) or that had *tolerance* values <0.2. As a result, we retained only 3 covariates (population size, percentage of rural population; and PT assistants part of the workforce, yes or no). Then, we progressively reintroduced into the shortened model, one by one, all the eliminated covariates (starting with those who had lowest *P* values in full model). If reintroducing one variable significantly improved the model fit (*r*^2^change yielding *P* values < .05), that covariate was retained. The process was repeated for all the covariates until we could no longer significantly improve the fit of model in progress. This whole process yielded 2 additional covariates (nominal gross national income [GNI], and current health expenditure as % of GDP) for the final model. Compared with the initial model, there were no significant differences in explained variance (*P *value of the *r*^2^change =.50). Hence, we only present the final model in the main results. The initial model is available on demand.

 For final model, homoscedasticity and normality assumptions were positively assessed respectively by plots of standardized residuals versus predicted values and a *Q-Q* plot. The Durbin Watson test did not support the presence of autocorrelation (*P* = .96). No item had low *tolerance* (all statistics above 0.5), and overall the model had a relatively good fit (r^2^ = 0.74; *P* < .01). Statistical significance was considered at *P* values < .05 for the main independent variable. Yet, for the 5 other covariates, 2 levels of statistical significance were applied: one with *P* values < .05 and another with *P* values of < .01; the latter accounts for a Bonferroni correction (0.05/5 = 0.01). The full statistical analysis was run using JASP 0.10.20 software.

## Results

 The supply of PTs and OTs per 10 000 population (mean = 15.7) varied widely across 35 high-incomes countries (SD = 10), ranging from 0.9 to 38.7, respectively for Trinidad and Tobago and Denmark (Table 2 and Figure 1).

**Table 2:** Descriptive Statistics for the Variables Included Into the Final Model

		**Mean**	**SD**	**Min.**	**Max.**
**Outcome variable**	*Supply* indicator (PTs and OTs per 10 000 population)	15.7	10	0.9	38.7
**Main independent variable**	*Need* indicator (YLD rates)	6442	1159	3345	8411
**Covariates**	GNI per capita	37 906	18 908	13 030	81 130
	Population size (in millions)	31.7	58.8	3.4	325
	Percentage of rural population	19.1%	11.6%	0%	49.8%
	Current health expenditure as % of GDP	9.0%	2.3%	4.5%	17.1%
	Categorial covariate	Yes		No	
	Physical therapy assistants, yes or no	40%		60%	

Abbreviations: GNI, gross national income; GDP, gross domestic product; PTs, physical therapists; OTs, occupational therapists; YLD, year lived with disability; SD, standard deviation.

 With respect to need for physical rehabilitation, the YLD Rates (mean = 6442; SD = 1159) ranged from 3345 in Panama to 8411 in Slovenia (Table 2 and Figure 1).

**Figure 2:**
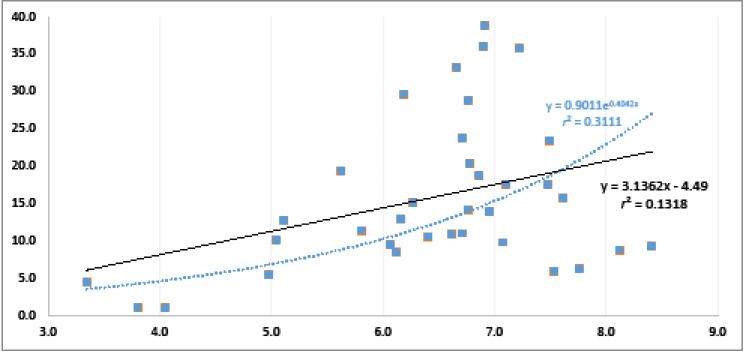
The Unadjusted Relationship Between PTs ans OTs Supply Per 10 000 People (*Y* Axis) and the Indicator of Physical Rehabilitation Need, ie, YLD Rates (in Thousands) for Rehabilitation-Sensitive Conditions (*X* Axis), for the 35 HICs Analyzed. Linear (black) and logarithmic (blue) regression models are shown for the data. Abbreviations: PTs, physical therapists; OTs, occupational therapists; YLD, year lived with disability; HICs, high-income countries.

Figure 1additionally shows that the growth in the supply does not reflect solely the growth in the need. For example, the 32 countries on the right-hand side all have ‘needs’ ranging between the 5.0 and 8.4 thousand YLDs (1.7 times the difference), but a supply ranging from 5.4 to 38.8 per 10 000 population (7.2 times the difference). Figure 2 provides a scatter plot and simple regression of the unadjusted relationship between need and supply indicators. Although significant at a 95% confidence level (*P* = .03), the relationship did not have a good fit in the linear regression (*r*^2^ = 0.13), and only slightly fitted better in a logarithmic type of regression (*r*^2^ = 0.31).

 Adjusting for the relevant covariates, the physical rehabilitation need was not a significant, independent predictor of the workforce supply (*P* = .11) (Table 3), which also happened in the initial model with all the covariates (*P* value = .25).

 The existence of PT assistants was a significant negative factor for the supply, at a 95% confidence level only (*P* = .03), while the percentage of rural population was marginally negatively related (*P* = .053) (Table 3). GNI per capita and current health expenditure in % of GDP were found to be positively related with the workforce supply, while population size was negatively related (all *P <*.01).

**Table 3:** Coefficients of the Final Multiple Regression Model for the Supply of PTs and OTs Per Population Size

	**Unstandardized** **Coefficient**	***T***	***P***	**95% CI**		**Tolerance**
				**Lower**	**Upper**	
Need indicator (YLD rates)	0.001	1.638	.113	-3.679e -4	0.003	0.851
GNI per capita	2.137e -4	3.540	.001^a^	9.005e -5	3.374e-4	0.743
Population size	-9.844e -8	-4.381	<.001^a^	-1.445e -7	-5.241e-8	0.526
Percentage of rural population	-0.192	-2.017	.053	-0.387	0.003	0.757
Current health expenditure as % of GDP	2.466	3.933	<.001^a^	1.182	3.750	0.453
Physical therapy assistants, yes or no	-4.507	-2.281	.030^b^	-8.555	-0.459	0.952

Abbreviations: PTs, physical therapists; OTs, occupational therapists; GNI, gross national income; GDP, gross domestic product; YLD, year lived with disability. ^a^ Statistically significant at a 99% confidence level (includes Bonferroni correction). ^b^ Statistically significant at a 95% confidence level (ie, without Bonferroni correction).

## Discussion

 The data did not support the hypothesis that physical rehabilitation need was independently associated to the workforce supply in HICs. However, after Bonferroni correction, other factors were associated to supply. Indeed, PT and OT supply was positively predicted by a lower population size, higher GNI per capita, or higher healthcare expenditure as percentage of GDP. Even though ‘needs-based’ workforce policies and planning have been increasingly advocated,^2,5-7,9,10,29,30^ our findings suggest that demographic and economic indicators are more significant determinants of PT and OT workforce supply in HICs than a composite indicator of physical rehabilitation need.

 Various factors may explain these findings. For instance, health workforce policies, planning, monitoring, and research activities were found rarely documented in the physical rehabilitation field.^3^ The few studies that map the rehabilitation workforce distribution within a country^31^ or forecast national workforce shortages/surplus,^25,26^ against supply requirements, have relied on ‘demand-based’ (eg, unfilled vacancies, insurance coverage, service utilization) rather than ‘needs-based’ indicators.^31-33^ Arguably, demand-side factors reflect pressing labor-market and economic forces,^25,26,31^ some of which this study have found relevant. Another explanation is that the composite indicator of physical rehabilitation need, derived from the GBD 2017, only became available in 2019.^14^ Previously, other indicators of need used in rehabilitation workforce studies have been based merely on demographic trends^30^ or on data from community health surveys.^34^ Finally, local and global stakeholders’ awareness has emerged only recently for the need to monitor, plan and develop rehabilitation resources, in tandem with the growing awareness of population ageing and the increasing rates of chronic conditions, disabilities, and corresponding physical rehabilitation needs.^3,14,20,35-38^

 Within the 35 HICs, great disparities were found for the OTs and PTs supply per capita, eg, up to 40 times the difference. Our inferential analysis showed that such a disparity was not explained by varying need levels; the latter had a more sensible variation, eg, up to 2.5 times the difference. We also found that the five Nordic countries are among the top six countries with the greatest OTs and PTs supply. Given that the level of government spending on healthcare proved not to be a significant covariate in this study, we speculate that the Nordic egalitarian culture, ethos and social policies, especially toward fulfilling the needs of the disadvantaged (eg, the elderly, people with disabilities), may have had an important role.^39,40^ For instance, the social, school-based (eg, for children with special needs), and broad welfare services in Nordic countries may create a high demand for therapists, beyond what the health sector (and public spending specifically in healthcare) could account for in the model.^41,42^ Whatever the reasons, the supply data convey that different countries/societies have substantially different numbers of population-adjusted PTs and OTs, for relatively equivalent levels of population need. This suggests substantial supply-need disparities across HICs.

 Although not directly analyzed, within-country disparities may have been latent factors. We found that having a larger population negatively influenced the supply of PTs and OTs per capita, while having a higher percentage of rural population was marginally related (*P* = .053). Together, these findings suggest that less densely populated areas, of countries with a vast population, may have a higher risk of not having enough PTs and OTs available. Such ecological trend is aligned with the Human Resource for Health literature in general,^27,43,44^ and that of the physical rehabilitation field in particular.^34,45,46^ For example, a study in the Province of Saskatchewan in Canada, using both need and PT supply indicators, found that the population that lived in rural areas were especially undersupplied compared to urban areas.^34^ Altogether, people living in rural areas in countries with a larger population, may be facing a double disparity, or double ecological risk, of facing needs-based shortages of PTs and OTs.

 Apart from the aggregated ecological trend across HICs, attention needs to be paid to individual countries and contexts. For instance, Singapore is representative of the “ecological fallacy” in our study, ie, inference based on aggregate data for the whole group does not necessarily apply to individual entities within the group.^47-49^ Indeed, Singapore has one of the highest (ie, seventh) GNIs per capita of the group, a population size about six times smaller than the average of the 35 HICs, and no rural population. Each of these the indicators, in the ecological trend, were associated to a higher PT and OT supply. But Singapore is amongst the least supplied, ie, fourth to last. This can be explained by the fact that Singapore only recently started PT and OT degree programs in a local university in 2016 and an accelerated professional conversion program in PT for mid-career locals in 2019.^16,50,51^ This finding hints to the need for complementing broad and large-scale ecological analysis with country-specific or nuanced, contextualized, and qualitative analyses of workforce data across fewer geographies^16^; it also highlights that countries with trends identified as at the odds with the ecological trend may be at a more pressing need for implementing deliberate workforce policies, which for example Singapore is recently doing so, ie, devising and implementing policy-driven expansion plans for the PTs and OTs workforce.^52^

 Finally, the official existence of PT assistants was negatively associated with the PT and OT supply at a 95% confidence level, although not after Bonferroni correction. This negative association is not surprising as the presence of assistant professions contributes to meet population needs in addition to, and often in complement of, the main professions; hence it may interfere with the demand for the latter.^28^ That can be an issue in the physical rehabilitation field, in which the practices and competencies of each type of rehabilitation professional can vary widely across countries, even HICs; and, for example, PT assistants in some countries perform roles that PTs typically perform in others where assistants do not exist officially.^16,20^ Although actual rehabilitation practices and competencies would be optimal in such a context, a practices- and competency-based workforce classification has not been established yet,^20^ and this makes that cross-location comparative workforce studies still needing to be based on professional labels.

###  Study Limitations

 This paper has several limitations:

 We had comparable, complete data only for 35 HICs; this reduced the statistical power and may have accounted for the inability to detect other relevant associations. Moreover, broad ecological trends may not apply to all individual HICs, as shown by the Singapore case. The trends should be understood only for the context of the HICs assessed, and not for the context of other countries, such as many LMICs – where large unmet rehabilitation needs exist and the development of the rehabilitation workforce is in its infancy.

 The indicator of the physical rehabilitation workforce supply only included data on PTs and OTs, while other professions (speech and language pathologists, rehabilitation-specialist physicians or nurses, orthotic and prosthetic professionals, etc) would be relevant as well.^3,10^ The results should not be inferred to represent physical rehabilitation more generally. In our data, proportions of PTs and OTs varied greatly from a 55%-45% PT-OT distribution (Israel) to 98%-2% (Italy), as the roles of these professions often partially overlap.^16^ In this study, we did not collect or analyze data on which roles do PT and OT professionals have in each country to understand these differences. Nonetheless, the fact the we have combined the supply of both professions into a composite supply indicator can fade the impact of these different distributions into the study’s results.

 In addition to data from other rehabilitation professions, we did not include data on generalist health professions, such as medical doctors or nurses, which could be used for comparative purposes. Yet, we suggest that this study on the historically neglected rehabilitation workforce can model others to examine whether need indicators are independent predictors of supply for other specific health workforces, or the health workforce overall. Also, unlike for PTs, we did not have an indicator on whether the OT supply per country was based on authoritative data or on an estimate. Moreover, the data for the PT and OT workforce supply was restricted to that reported from national associations of the respective professions to the international federations or confederations regarding the number of practicing professionals, while other mechanisms (eg, labor market surveys) and broader supply data (eg, the whole stock of professionals available, even unemployed) could, if available, also be informative as well as add robustness to the findings. Also, we did not had data pointing for the percentage of PTs or OTs working in the public or private sector. It is noteworthy, though, that items on the level of domestic government spending in healthcare did not prove to be significant factors in this study.

 The composite indicator of need was based on current data extracted from the GBD 2017, the largest global epidemiological study to date; however, that data was based on diseases and their sequalae, while physical rehabilitation addresses functional limitations which account for environmental factors as well. Similarly, the set of conditions used for computing the need indicator was based, for example, in the findings of existing systematic reviews on the effectiveness of the physical rehabilitation interventions^14^; however, that set of conditions cannot be considered a fixed standard and can evolve with scientific advances.

## Conclusion

 PT and OT workforce supply is highly variable across HICs. This variability is not accounted for by a composite indicator of population need but rather by financial factors and population size. These broad ecological findings, along with country-specific data, should inform the development of deliberate physical rehabilitation workforce policies and planning, whether needs-based or not.

## Ethical issues

 All data used are in the public domain.

## Competing interests

 Authors declare that they have no competing interests.

## Authors’ contributions

 TJ: Conception and design; acquisition of data; analysis and interpretation of data; drafting the manuscript; critical revision of the manuscript for important intellectual content; statistical analysis. ML: Conception and design; analysis and interpretation of data; critical revision of the manuscript for important intellectual content. HH: Conception and design; analysis and interpretation of data; critical revision of the manuscript for important intellectual content. GD: Analysis and interpretation of data; critical revision of the manuscript for important intellectual content. GK: Analysis and interpretation of data; critical revision of the manuscript for important intellectual content. IF: Conception and design; analysis and interpretation of data; critical revision of the manuscript for important intellectual content; statistical analysis.

## Authors’ affiliations

^1^Global Health and Tropical Medicine & WHO Collaborating Center on Health Workforce Policy and Planning, Institute of Hygiene and Tropical Medicine - NOVA University of Lisbon, Lisbon, Portugal. ^2^School of Medicine, Duke University, Durham, NC, USA. ^3^Duke Global Health Institute, Duke University, Durham, NC, USA. ^4^Physical Medicine and Rehabilitation Service, Durham Veterans Administration Medical Center, Durham, NC, USA. ^5^Division of Geriatrics, Department of Medicine, Duke University Medical Center, Durham, NC, USA. ^6^Saw Swee Hock School of Public Health, National University of Singapore, Singapore, Singapore.

## Supplementary file


Supplementary file 1. Raw Data (Extracted) Per Country.

## References

[1] Kuhlmann E, Batenburg R, Dussault G (2018). A people-centred health workforce in Europe: how to make it happen?. Health Policy.

[2] Campbell J, Buchan J, Cometto G (2013). Human resources for health and universal health coverage: fostering equity and effective coverage. Bull World Health Organ.

[3] Jesus TS, Landry MD, Dussault G, Fronteira I (2017). Human resources for health (and rehabilitation): Six Rehab-Workforce Challenges for the century. Hum Resour Health.

[4] Cometto G, Buchan J, Dussault G (2020). Developing the health workforce for universal health coverage. Bull World Health Organ.

[5] Tomblin Murphy G, Birch S, MacKenzie A, Rigby J (2016). Simulating future supply of and requirements for human resources for health in high-income OECD countries. Hum Resour Health.

[6] World Health Organization (WHO). Global Strategy on Human Resources for Health: Workforce 2030. Geneva, Switzerland; WHO; 2016.

[7] Kuhlmann E, Batenburg R, Wismar M (2018). A call for action to establish a research agenda for building a future health workforce in Europe. Health Res Policy Syst.

[8] Scheffler RM, Campbell J, Cometto G (2018). Forecasting imbalances in the global health labor market and devising policy responses. Hum Resour Health.

[9] Ono T, Lafortune G, Schoenstein M. Health Workforce Planning in OECD Countries: A Review of 26 Projection Models from 18 Countries. OECD Health Working Papers, No. 62. Paris: OECD; 2013. 10.1787/5k44t787zcwb-en

[10] MacKenzie A, Tomblin Murphy G, Audas R (2019). A dynamic, multi-professional, needs-based simulation model to inform human resources for health planning. Hum Resour Health.

[11] World Health Organization (WHO). Rehabilitation 2030: A Call for Action. The Need to Scale Up Rehabilitation. Geneva, Switzerland: WHO; 2017.

[12] Prince MJ, Wu F, Guo Y (2015). The burden of disease in older people and implications for health policy and practice. Lancet.

[13] Chatterji S, Byles J, Cutler D, Seeman T, Verdes E (2015). Health, functioning, and disability in older adults--present status and future implications. Lancet.

[14] Jesus TS, Landry MD, Hoenig H (2019). Global need for physical rehabilitation: systematic analysis from the Global Burden of Disease Study 2017. Int J Environ Res Public Health.

[15] Gupta N, Castillo-Laborde C, Landry MD (2011). Health-related rehabilitation services: assessing the global supply of and need for human resources. BMC Health Serv Res.

[16] Jesus TS, Koh G, Landry M (2016). Finding the “right-size” physical therapy workforce: international perspective across 4 countries. Phys Ther.

[17] Landry MD, Ricketts TC, Fraher E, Verrier MC (2009). Physical therapy health human resource ratios: a comparative analysis of the United States and Canada. Phys Ther.

[18] Tomblin Murphy G, MacKenzie A, Alder R, Langley J, Hickey M, Cook A (2013). Pilot-testing an applied competency-based approach to health human resources planning. Health Policy Plan.

[19] Liu JX, Goryakin Y, Maeda A, Bruckner T, Scheffler R (2017). Global health workforce labor market projections for 2030. Hum Resour Health.

[20] Jesus TS, Landry MD, Dussault G, Fronteira I (2019). Classifying and measuring human resources for health and rehabilitation: concept design of a practices- and competency-based international classification. Phys Ther.

[21] World Physiotherapy. Profiling the profession: WCPT’s data collection. https://world.physio/our-members. Accessed July 20, 2019. Updated 2019.

[22] World Federation of Occupational Therapists (WFOT). Occupational Therapy Human Resources Project 2018 (Edited) – Alphabetical. WFOT; 2018. https://www.wfot.org/resources/2018-occupational-therapy-human-resources-project-edited-alphabetical. Accessed July 28, 2019.

[23] (2018). Population and fertility by age and sex for 195 countries and territories, 1950-2017: a systematic analysis for the Global Burden of Disease Study 2017. Lancet.

[24] (2018). Global, regional, and national incidence, prevalence, and years lived with disability for 354 diseases and injuries for 195 countries and territories, 1990-2017: a systematic analysis for the Global Burden of Disease Study 2017. Lancet.

[25] McPake B, Maeda A, Araújo EC, Lemiere C, El Maghraby A, Cometto G (2013). Why do health labour market forces matter?. Bull World Health Organ.

[26] Sousa A, Scheffler RM, Nyoni J, Boerma T (2013). A comprehensive health labour market framework for universal health coverage. Bull World Health Organ.

[27] Cosgrave C, Malatzky C, Gillespie J (2019). Social determinants of rural health workforce retention: a scoping review. Int J Environ Res Public Health.

[28] Maier CB, Batenburg R, Birch S, Zander B, Elliott R, Busse R (2018). Health workforce planning: which countries include nurse practitioners and physician assistants and to what effect?. Health Policy.

[29] Organisation for Economic Co-operation and Development (OECD). Health Workforce. Paris: OECD; 2019. https://www.oecd.org/health/health-systems/workforce.htm. Accessed September 29, 2019.

[30] Christiansen M, Schmidt JP, Shkel D, Kaluscha R, Tepohl L, Krischak G (2018). A projection of the need for rehabilitation in Germany till 2040 based on demographic factors. Gesundheitswesen.

[31] Shah TI, Milosavljevic S, Trask C, Bath B (2019). Mapping physiotherapy use in Canada in relation to physiotherapist distribution. Physiother Can.

[32] Landry MD, Hack LM, Coulson E (2016). Workforce projections 2010-2020: annual supply and demand forecasting models for physical therapists across the United States. Phys Ther.

[33] Lin V, Zhang X, Dixon P (2015). Occupational therapy workforce in the United States: forecasting nationwide shortages. PM R.

[34] McFadden B, Jones McGrath K, Lowe T (2016). Examining the supply of and demand for physiotherapy in Saskatchewan: the relationship between where physiotherapists work and population health need. Physiother Can.

[35] Skempes D, Melvin J, von Groote P, Stucki G, Bickenbach J (2018). Using concept mapping to develop a human rights based indicator framework to assess country efforts to strengthen rehabilitation provision and policy: the Rehabilitation System Diagnosis and Dialogue framework (RESYST). Global Health.

[36] Krug E, Cieza A (2017). Strengthening health systems to provide rehabilitation services. Bull World Health Organ.

[37] Gutenbrunner C, Nugraha B, Gimigliano F, Meyer T, Kiekens C (2020). International Classification of Service Organization in Rehabilitation: an updated set of categories (ICSO-R 20). J Rehabil Med.

[38] World Health Organization (WHO). Rehabilitation in Health Systems: Guide for Action. Geneva: WHO; 2019.

[39] Burau V, Carstensen K, Lou S, Kuhlmann E (2017). Professional groups driving change toward patient-centred care: interprofessional working in stroke rehabilitation in Denmark. BMC Health Serv Res.

[40] Mackenbach JP (2012). The persistence of health inequalities in modern welfare states: the explanation of a paradox. Soc Sci Med.

[41] Fortune N, Madden R, Almborg AH (2018). Use of a new international classification of health interventions for capturing information on health interventions relevant to people with disabilities. Int J Environ Res Public Health.

[42] Johansen H, Østlie K, Andersen L, Rand-Hendriksen S (2015). Adults with congenital limb deficiency in Norway: demographic and clinical features, pain and the use of health care and welfare services A cross-sectional study. Disabil Rehabil.

[43] Rechel B, Džakula A, Duran A (2016). Hospitals in rural or remote areas: an exploratory review of policies in 8 high-income countries. Health Policy.

[44] Hempel S, Gibbons MM, Ulloa JG, et al. Rural Healthcare Workforce: A Systematic Review. Washington, DC: Department of Veterans Affairs; 2015. 28121089

[45] Cosgrave C, Maple M, Hussain R (2018). An explanation of turnover intention among early-career nursing and allied health professionals working in rural and remote Australia - findings from a grounded theory study. Rural Remote Health.

[46] Wilson RD, Lewis SA, Murray PK (2009). Trends in the rehabilitation therapist workforce in underserved areas: 1980-2000. J Rural Health.

[47] Bryere J, Pornet C, Copin N (2017). Assessment of the ecological bias of seven aggregate social deprivation indices. BMC Public Health.

[48] Sedgwick P (2015). Understanding the ecological fallacy. BMJ.

[49] Zeoli AM, Paruk JK, Pizarro JM, Goldstick J (2019). Ecological research for studies of violence: a methodological guide. J Interpers Violence.

[50] Davie S. New Degree Courses to Raise SIT Intake by 300. The Straits Times; 2015.

[51] Singapore Institute of Technology. New Accelerated Professional Conversion Programme in Physiotherapy. https://www.singaporetech.edu.sg/digitalnewsroom/new-accelerated-professional-conversion-programme-in-physiotherapy/. Accessed February 27, 2020. Published 2019.

[52] Ministry of Health. Speech by Chan Heng Kee, Permanent Secretary, Ministry of Health, At the Launch of the Temasek Foundation iMOVE Programme. Singapore: Ministry of Health; 2019. https://www.moh.gov.sg/news-highlights/details/speech-by-chan-heng-kee-permanent-secretary-ministry-of-health-at-the-launch-of-the-temasek-foundation---imove-programme-6-may-2019-at-alexandra-hospital. Accessed February 27, 2020.

